# Stearoyl-CoA Desaturase-1 dependent lipid droplets accumulation in cancer-associated fibroblasts facilitates the progression of lung cancer

**DOI:** 10.7150/ijbs.74924

**Published:** 2022-10-18

**Authors:** Yana Zhang, Zhuoyu Gu, Jiajia Wan, Xiaohan Lou, Shuangqing Liu, Yuan Wang, Yangyang Bian, Fei Wang, Zhenzhen Li, Zhihai Qin

**Affiliations:** 1 Medical Research Center, The First Affiliated Hospital of Zhengzhou University, Zhengzhou, Henan 450052, China.; 2 Henan International Joint Laboratory of Tumor Immune Microenvironment, Zhengzhou, Henan 450052, China.; 3 Key Laboratory of Protein and Peptide Pharmaceuticals, Institute of Biophysics, Chinese Academy of Sciences, Beijing 100101, China.; 4 College of Life Science, Northwest University, Xi'an, Shanxi 710069, China.

**Keywords:** Lung Cancer, Cancer-associated Fibroblasts, Lipid Droplets, Hypoxia-Inducible Factor-1α, SCD1

## Abstract

**Rationale:** Cancer-associated fibroblasts (CAFs) are the main components in the tumor microenvironment (TME) and facilitate lung cancer progression. Studies have reported that metabolic reprogramming can regulate the function of CAFs, especially abnormal lipid metabolism. Lipid droplets (LDs) are ubiquitous organelles that store neutral lipids and have a crucial role in lipid metabolism. However, little is known about the synthesis and functions of LDs in lung CAFs.

**Methods:** *TetO-EGFR^L858R^; CCSP-rtTA* transgenic mouse model was used to establish a spontaneous pulmonary tumor model and investigate the accumulation of LDs in CAFs. The effect of LDs accumulation on the phenotype change of fibroblasts was estimated *in vitro* using mouse fibroblast cell lines. RNA sequencing, Western blotting, RT-PCR, and DNA-pull down were performed to determine the mechanism of LDs synthesis in fibroblasts.

**Results:** We found that LDs were enriched in lung CAFs and induced the pro-tumoral phenotype of CAFs with increased expression of α-smooth muscle actin (α-SMA) and Collagen alpha-2 (I) chain (COL1A2). As the main regulator, hypoxia-inducible factor-1α (HIF-1α) was highly expressed in activated fibroblasts and increased the content of LDs. RNA-sequencing results showed that Stearoyl-CoA Desaturase1 (SCD1) was a downstream gene of HIF-1α, which upregulated the number of LDs in fibroblasts. Importantly, SCD1 inhibition reduced the growth of lung tumors, which was correlated with LDs decrease in CAFs. Analysis of human lung adenocarcinoma tissue chip revealed that CAFs with a high level of SCD1 were positively correlated with the expression of HIF-1α and poor survival in lung cancer patients.

**Conclusions:** The HIF-1α/SCD1 axis regulates the accumulation of LDs in CAFs, which might represent a novel target for lung cancer therapy.

## Introduction

The incidence of lung cancer is second only to breast cancer, while it is the leading cause of cancer-related deaths; an estimated 1.8 million deaths were caused by lung cancer in 2020 1. Studies have shown that the behavior of cancers is determined by both cancer cells and the tumor microenvironment (TME) 2. Cancer-associated fibroblasts (CAFs) are one of the most important components of the TME and show a high expression of several proteins such as α-smooth muscle actin (α-SMA), fibroblast-activating protein (FAP), collagen alpha-2 (I) chain (COL1A2), and podoplanin (PDPN) 3, 4. Because of the non-specific markers, CAFs comprise many sub-types with different functions 3, 5. The functions of CAFs are strongly related to the progression of lung cancer 6. For example, during the progression of lung cancer, CAFs regulate the synthesis and remodeling of the extracellular matrix and the production of growth factors, which increase the stiffness of tumor tissue and tumor growth 7, 8. CAFs-mediated paracrine transforming growth factor-β (TGF-β) signaling leads to tumor heterogeneity in lung cancer 9. Besides, CAFs decrease the chemosensitivity of lung cancer cells by inhibiting cisplatin-induced apoptosis 10. However, many studies have shown that the functions of CAFs are determined by the different subtypes 11, which makes it challenging to study CAFs.

During tumor progression, the metabolic reprogramming of CAFs not only provides energy for tumor cells, but also improves the survival of themselves in the TME 12. For example, CAFs can promote cancer progression by secreting growth factors and amino acids 13. Besides, CAFs release metabolic intermediates such as lactic acid and L-Glutamine, which can be utilized by tumor cells 14, 15. Many studies have shown that the metabolic reprogramming of CAFs affects the behaviors of tumor cells 16, but how metabolic reprogramming directly influences the function of CAFs is unclear. It is well-known that chronic hypoxia can induce epigenetic reprogramming of glycolysis in fibroblasts to protect them from a poor TME 17. Lipid metabolism is the main part of metabolism and is essential for the maintenance of the malignant microenvironment 16. As a part of lipid metabolism, lipid droplets (LDs) are storage organelles at the center of lipid and energy homeostasis and play important roles in regulating the cell phenotypes. Studies have shown that the increased number of LDs is positively correlated with the stemness of breast cancer cells 18. Besides, sodium oleate induces the accumulation of LDs and regulates the suppressive phenotype of myeloid cells 19. Inhibiting the formation of LDs causes the transformation of the phenotype of macrophages from M2 to M1 20. However, there is no study focusing on the effects of LDs on CAFs in lung cancer.

The synthesis of LDs includes the conversion of excess free fatty acids (FAs) into neutral triglycerides (TAGs) and their storage in LDs, which can serve as the main source of energy 21, 22. The regulation of FA uptake, synthesis, storage, and usage depends on hypoxia-inducible factor-1α (HIF-1α) 23. HIF-1α-mediated metabolic reprogramming is involved in a variety of tumor processes including lung cancer progression 24, 25. The complex environment in tumors also influences the expression of HIF-1α in a variety of stromal cells in the TME 24, 26. Our previous study demonstrated that hypoxia, TGF-β1, and tumor conditioned medium (CM) increase the expression of HIF-1α, which plays an important role in the activation of CAFs 8. However, little is known about the connection between HIF-1α and the accumulation of LDs in CAFs.

Here, we identified HIF-1α as a novel SCD1 promoter-binding protein that promotes the accumulation of LDs in fibroblasts. Additionally, the high content of LDs induces the upregulation of α-SMA and COL1A2, which in turn activate fibroblasts. Targeting the HIF-1α/SCD1 axis in CAFs might be a promising strategy for lung cancer therapy.

## Methods

### Tumor models and treatments

All mice were kept in specific pathogen-free conditions and conducted with approval from the First Affiliated Hospital of Zhengzhou University Ethics Committee. The transgenic mice (*TetO-EGFR^L858R^; CCSP-rtTA*) were obtained from Professor Lin Xin of Tsinghua University and described previously 27. To induce the growth of lung tumors, doxycycline (1 mg/mL) was administered to the mice through drinking water at five weeks of age. The water was changed every two days for 3.5 months. After the mouse lung cancer model was established, the SCD1 inhibitor A939572 (20 mg/kg) or vehicle was administered through oral gavage daily for 14 days.

For mice experiments, Mouse Lewis lung cancer (LLC) cells (1×10^5^/mouse) were co-injected with or without fibroblasts (1×10^6^/mouse; NIH 3T3 or MEF) subcutaneously into C57 mice (Beijing Charles River Laboratory Animal Company). From the eighth day, tumor growth was monitored every two days, and tumor volume was calculated as length×width×width/2.

### Cells isolation and culture

Mouse Lewis lung cancer (LLC) cells were obtained from Professor Li Yan of the Academy of Military Medical Sciences. MEF cells (immortalized MEF cells) were a gift from Prof. Lin Xin of Tsinghua University. The NIH 3T3 cell line was obtained from ATCC (Manassas, VA, USA) and cultured with Dulbecco's modified eagle medium (DMEM) containing 10% fetal bovine serum (FBS, ST30-3302, PAN-Biotech GmbH). The LLCs were transfected with a lentivirus green fluorescence protein (GFP) according to previously described method 28. Mouse primary lung CAFs and normal fibroblasts (NFs) were isolated from the mouse lung cancer model (*TetO-EGFR^L858R^;CCSP-rtTA*) as previously described 8. The cultured CAFs and matched NFs were used after five passages following primary culture. After 72 h of incubation, the conditioned medium of the LLC cells (CM) was collected and centrifuged at 16,000 g for 5 min to remove cell debris. The cells were cultured at 37 °C, 5% CO_2_, and 20% O_2,_ except for those with special instructions. The hypoxic condition was 37 °C, 1% O_2_, 5% CO_2_, and 94% N_2_.

### H&E staining and Masson staining

The tissue sections were deparaffined and rehydrated as described previously 29. After routine processing, lung tissue sections (6 µm thick) were stained with H&E for histological analysis and Masson staining for collagen deposition analysis.

### Oil Red O Staining

Frozen sections from mice subcutaneous tumors and the slides with cells were fixed in 4% formalin for 20 min, then washed with 60% propylene glycerol. The sections were stained with 0.5% Oil Red O (Sigma, St. Louis, Missouri, USA) in propylene glycerol for 10 min at 60 °C, and the cells were stained for 5 min at room temperature. Nuclei were counterstained with hematoxylin for bright-field microscopic examination. The red LDs were visualized by microscopy, and the fluorescence intensity was analyzed by using Image J.

### Tissue and cell immunofluorescence

OCT-embedded samples were sectioned, and the frozen slides or cells were fixed with 4.0% paraformaldehyde and incubated for 15 min at room temperature. The sections were blocked with 5% bovine serum albumin in PBST (tween-20) and incubated with primary antibodies overnight at 4 °C. The primary antibodies used included goat anti-α-SMA (Abcam, Cat# Ab21027), rabbit anti-s100a4 (Abcam, Cat# Ab27957), rabbit anti-ADRP (Proteintech, Cat#15294-I-AP) and rabbit anti-Proliferating cell nuclear antigen (PCNA) (Proteintech, Cat#10205-I-AP). The slides were incubated with the immunofluorescent secondary antibody for 0.5 h at room temperature in the dark. A tissue microarray (HLugA180Su07) was purchased from Shanghai Outdo Biotech Company. For the tissue microarray, it was de-waxed and heat-induced for antigen retrieval. The tissue microarray was incubated with anti-α-SMA (CST, Cat# 56856S), anti-HIF-1α (Abcam, Cat# ab179483) and anti-SCD1 (CST, Cat# 2794S); the PPD-540 and PPD-620 digital image system (PANOVUE, China) were used for visualization. The nuclei were stained using Fluoro-Gel II 20 with DAPI (Electron Microscopy Sciences, USA), and the slides were examined by fluorescence microscopy.

### Flow cytometry sorting

Single-cell suspensions prepared directly from lung cancer tissues and normal lung tissues were stained with PE-labelled anti-EpCAM (clone G8.8), APC-labelled anti-CD31 (clone MEC13.3), and Alexa Fluor 700-labelled anti-CD45 (clone I3/2.3). The dead cells were labelled with DAPI, and the LDs were labelled with BODIPY®493/503. All antibodies were purchased from Biolegend (California, USA). The cells were analyzed using FACSCanto II (BD Biosciences, San Diego, USA).

## Materials and reagents

Cobalt chloride (Cocl2) and PF-06424439 (inhibitor of DGAT2) were obtained from Sigma-Aldrich. BODIPY®493/503, LipidTOX™ neutral lipid stain, and Lipofectamine 3000 were purchased from Invitrogen (California, USA). TGF-β1 was obtained from Pepro Tech (Rocky Hill, USA). The inhibitors A939572 (inhibitor of SCD1), A922500 (inhibitor of DGAT1), and JZL-184 (inhibitor of MAGL) were obtained from MedChemExpress (New Jersey, USA).

### BODIPY®493/503 Staining

Cells were washed with PBS and labeled with BODIPY®493/503 at 37 °C for 15 min. Then, the cells were digested and analyzed using the FACS Canto II device (BD Biosciences).

### HCS LipidTOX™ neutral lipid stains

Cells were fixed with 4.0% formaldehyde and incubated for 30 min. Formaldehyde was removed, and then the cells were labeled with the neutral lipid staining solution. The 1,000× LipidTOX™ neutral lipid stain (Invitrogen) was diluted 1:1,000. The plates were sealed at room temperature for 30 min before imaging.

### Real-time quantitative polymerase chain reaction (RT-qPCR)

Total RNA was extracted using RNAiso plus (TAKARA; #1089527), according to a previously described protocol, and reverse transcribed using PrimeScript RT reagent kit with gDNA Eraser (TAKARA; #RR047A). Real-time qPCR was performed using SYBR GreenTM Primix Ex TaqTM II (TAKARA; #RR820A) in Step One® sequence detection system (Applied Biosystems, Milan, Italy). Each sample was examined at least in triplicate. The relative RNA expression was calculated using the 2^-∆∆Ct^ method. The primers used are presented in the supplementary data (Table S1). The gene of the ribosomal protein 18S was used as a control gene to obtain normalized values.

### Western blot analysis

The cultured cells were lysed in RIPA buffer with 1% PMSF, 2% protease, and phosphatase inhibitors for 30 min, and the protein concentrations were measured with the BCA kit. The lysates were fractionated by 10% SDS-PAGE and transferred onto a nitrocellulose membrane. The following primary antibodies were used: anti-α-SMA (Abcam, ab21027; diluted 1:1,000), anti-COL1A2 (ProteinTech; 14695-I-AP; diluted 1:200), anti-HIF-1α (R&D Systems; Mab1536; diluted 1:1,000), anti-SCD1 (Origene, TA309938; diluted 1:1,000), and anti-β-Actin (Abclonal, AC006; diluted 1:10,000). Thereafter, the membranes were incubated with the corresponding secondary Horseradish-peroxidase-conjugated (HRP) conjugated antibodies. The signals were detected using the western ECL substrate (ConWin Biotech, Beijing, China).

### Adenovirus Construction, Purification, and Infection

The HIF-1α (NM_001313919.1) knockout fibroblasts (NIH 3T3 and MEF cells) were constructed and stored in our laboratory as described previously 8. SCD1-overexpressed (NM_009127.4) lentiviral fluid was purchased from Genechem Company (Shanghai). Cells were transfected with the lentiviral fluid, and the stable clones were selected after two weeks using 3 µg/mL puromycin.

### RNA-sequencing analysis

The expression of HIF-1α was knocked out in NIH 3T3 by CRISPR Cas9 as indicated before 8. Total RNA was extracted by using Trizol (Invitrogen, Carlsbad, CA, USA) and subjected to RNA-sequencing analysis by Beijing Genomics institution (BGI, Shenzhen, China). The raw transcriptomic reads were mapped to a reference genome and the gene expression levels by using BGI Dr.Tom 3.0. Significantly differentially expressed genes were acquired by setting log 2fold change >0.0.

### Promoter reporters and Dual-luciferase assay

A fragment containing the core promoter region of mSCD1 was inserted between the xHOI and kpnІ sites of the firefly luciferase vector pGL4.10, and the Renilla luciferase control reporter vector pRL-TK was used as a control. The cells (2×10^4^ cells/well) were seeded in a 96-well plate. After the cell confluence reached 70%, pGL4.10-hTERT-85 and pRL-TK were transfected into 3T3-MOCK and 3T3-OVER cells with Lipofectamine 3000 (Invitrogen, Carlsbad, CA). Dual-luciferase assay was performed using the Dual-Luciferase® Reporter Assay System (Promega, Madison, WI).

### Streptavidin-agarose pulldown assay

The mSCD1 promoter-binding proteins were analyzed by the streptavidin-agarose pulldown assay as described previously 30. Briefly, nuclear protein extracted from NIH 3T3 cells was incubated with 10 µg of biotin-labeled double-stranded DNA probes corresponding to the nucleotide -85 to +197 of the mSCD1 promoter region and 100 µL of streptavidin-agarose beads at 4 °C overnight. The mixture was then centrifuged at 500 g. The DNA-protein complex was fractionated by 10% SDS-PAGE and incubated with an anti-HIF-1α primary antibody. The bound proteins were visualized by the western ECL substrate.

### Data analysis

All data are presented as the Mean ± SD using GraphPad Prism V 7.0. Comparisons between two groups were calculated using unpaired Student's t test and comparisons of more than two groups were determined using one-way ANOVA Tukey's multiple comparisons. The survival analysis was determined by the Kaplan-Meier plotter. Differences between groups with p-values lesser than 0.05 were considered to be statistically significant.

## Results

### Accumulation of LDs in lung CAFs

**Figure 1:**
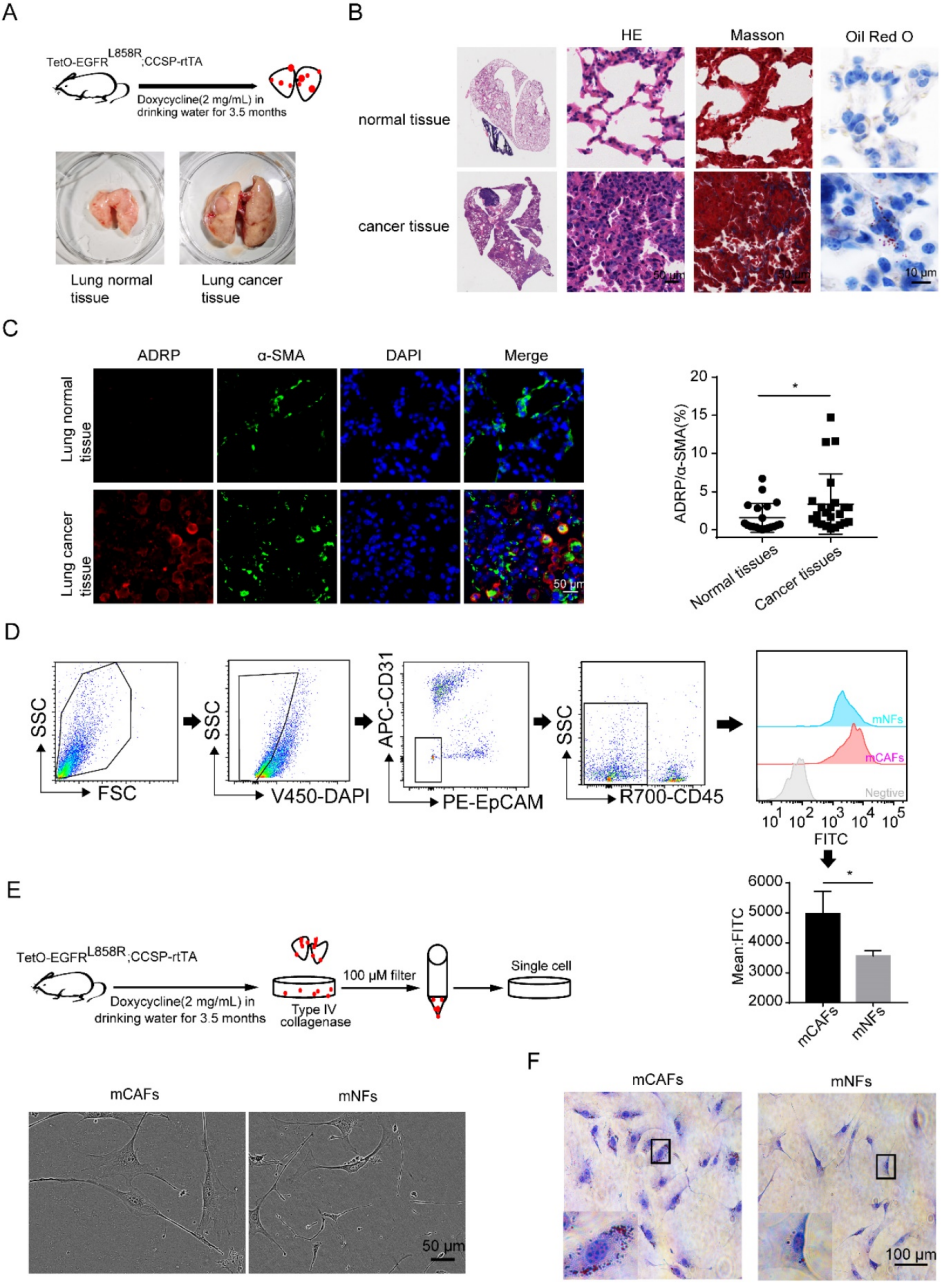
** Accumulation of LDs in lung CAFs. (A)** The doxycycline-induced mouse lung cancer model (*TetO-EGFR^L858R^; CCSP-rtTA*) was established (N = 5). **(B)** Representative images of mouse lung cancer tissues and normal lung tissues after H&E staining, Masson staining and Oil Red O staining (Scale bar of H&E and Masson: 50 µm; Scale bar of oil red O: 10 µm). **(C)** Representative immunofluorescence images of ADRP (red) co-stained with α-SMA (green) in frozen sections of mouse lung cancer tissues and normal lung tissues (DAPI: blue; Scale bar: 50 µm). **(D)** The LD contents of CAFs and NFs (DAPI-/CD45-/CD31-/EpCAM-) were detected by BODIPY staining (FITC) (N = 5). **(E)** The morphology of mCAFs and mNFs isolated from doxycycline-induced mouse lung cancer model (*TetO-EGFR^L858R^; CCSP-rtTA*) under white light (Scale bar: 50 µm, N = 3). **(F)** The Oil Red O staining of mCAFs and mNFs (Scale bar: 100 µm). Data are shown as the Mean±SD; *p < 0.05.

The mouse lung cancer model (*TetO-EGFR^L858R^; CCSP-rtTA*) was established using doxycycline hydrochloride (Figure 1A) 31. The results of H&E staining showed that the normal lung epithelial cells were replaced by a jumble of tumor cells with hyperchromatic nuclei. Besides, there was more collagen deposition and accumulation of LDs in the lung cancer tissues compared to that in the normal lung tissues, as demonstrated by Masson and Oil Red O staining (Figure 1B). Adipose differentiation-related protein (ADRP) is an LD protein found in most cells and tissues 19. By co-staining ADRP and α-SMA, we found that lung CAFs were enriched with LDs (Figure 1C). Additionally, CAFs and NFs (DAPI^-^/CD45^-^/CD31^-^/EPCAM^-^ cells) were analyzed by flow cytometry to confirm the higher enrichment of LDs in CAFs (Figure 1D).

To further confirm the above findings, the primary CAFs and NFs were isolated and cultured *in vitro* (Figure 1E). CAFs were identified with higher expression of α-SMA and S100A4 compared to their expression in NFs (Figures S1A and S1B), which was similar to the results found in other studies 3. Oil Red O staining revealed that more LDs were also observed in cultured CAFs compared to that in NFs (Figure 1F). These findings suggested that the accumulation of LDs might be one of the characteristics of CAFs, which is independent of the *in vivo* or *in vitro* environment.

### The accumulation of LDs is involved in the regulation of fibroblast activation

**Figure 2:**
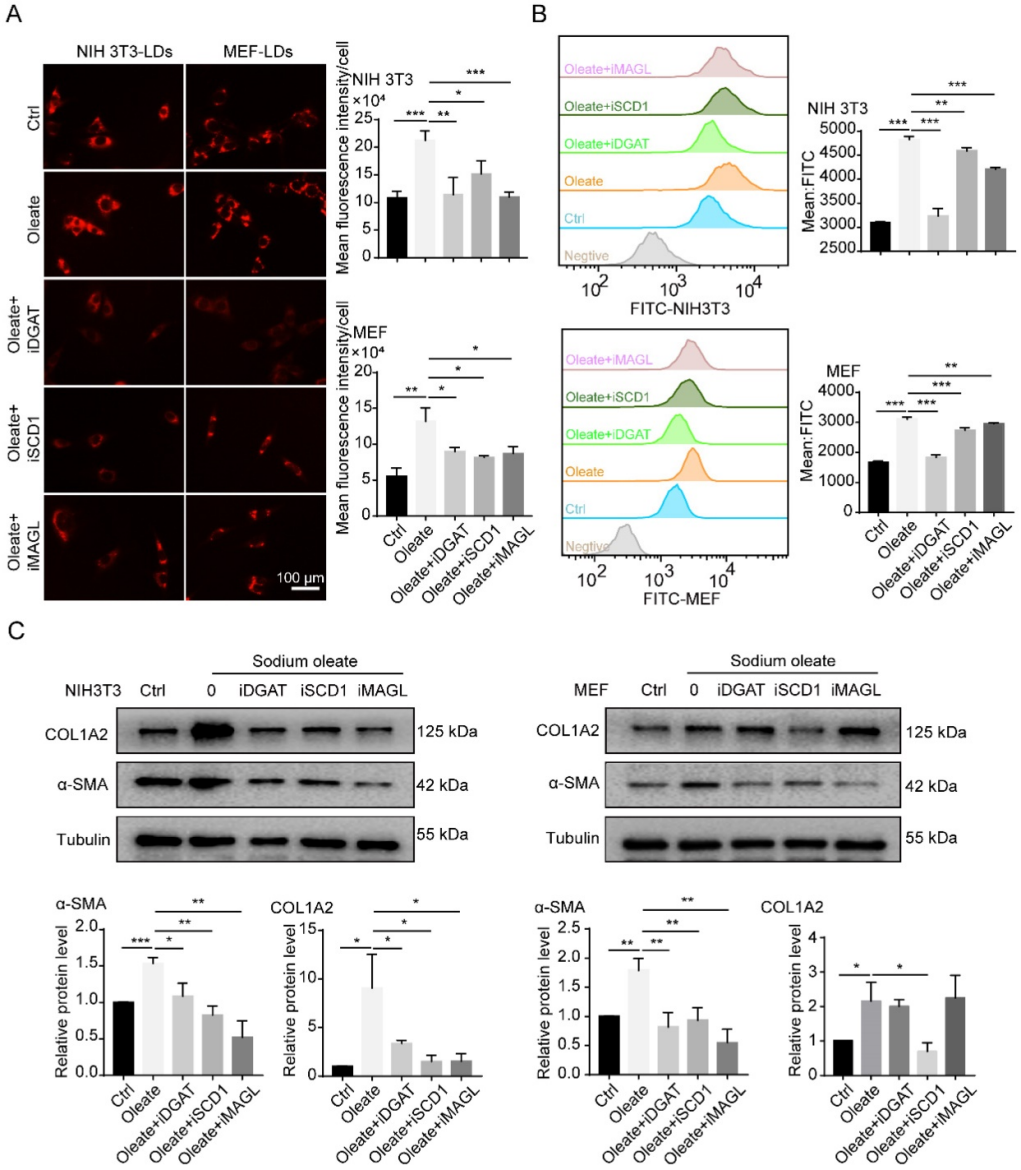
** The accumulation of LDs is involved in the regulation of fibroblast activation. (A)** The lipid-tox red staining of fibroblasts (NIH 3T3 and MEF) that were treated with/without sodium oleate (50 µM) and the LDs inhibitors (iDGAT: iDGAT1-10 µM+ iDGAT2-10 µM, iSCD1-100 nM, iMAGL-20 nM; Scale bar: 100 µm) for 12h (N = 5). **(B)** The BODIPY staining (FITC) of fibroblasts (NIH 3T3 and MEF) that were treated with/without sodium oleate (50 µM) and the LDs inhibitors (iDGAT, iSCD1 and iMAGL) for 12h (N = 3). **(C)** The protein expression of α-SMA and COLlA2 in fibroblasts (NIH 3T3 and MEF) that were treated with/without sodium oleate (50 µM) and the LDs inhibitors (iDGAT, iSCD1 and iMAGL) for 24h (N = 3). Data are shown as the Mean±SD; *p < 0.05, ** *p*< 0.01, ***p < 0.001.

CAFs are considered to be the activated fibroblasts in the TME 2. To further understand the effect of LDs on CAFs, we investigated the relationship between the accumulation of LDs and fibroblast activation. Sodium oleate was reported to induce lipid synthesis in cultured fibroblasts 19. In line with expectations, sodium oleate significantly upregulated the number of LDs in fibroblasts and increased the expression of COL1A2 and α-SMA (Figures 2A-2C). During the formation of LDs, SCD1 is responsible for the synthesis of unsaturated fatty acids, including palmitoleic (C16:1 n-7) and oleic (C18:1 n-9) acids, while diglyceride acyltransferase (DGAT) controls the transformation of free FAs into LDs and monoacyglycerol lipase (MAGL) mainly plays an important role in the export of LDs into the cytoplasm 19. When the catalytic activities of SCD1, DGAT and MAGL were blocked, the synthesis of LDs was inhibited in fibroblasts (Figures 2A and 2B), along with the expression of α-SMA (Figure 2C). These results indicated that the accumulation of LDs might regulate the activation of fibroblasts.

### HIF-1α is necessary for the accumulation of LDs in fibroblasts

**Figure 3:**
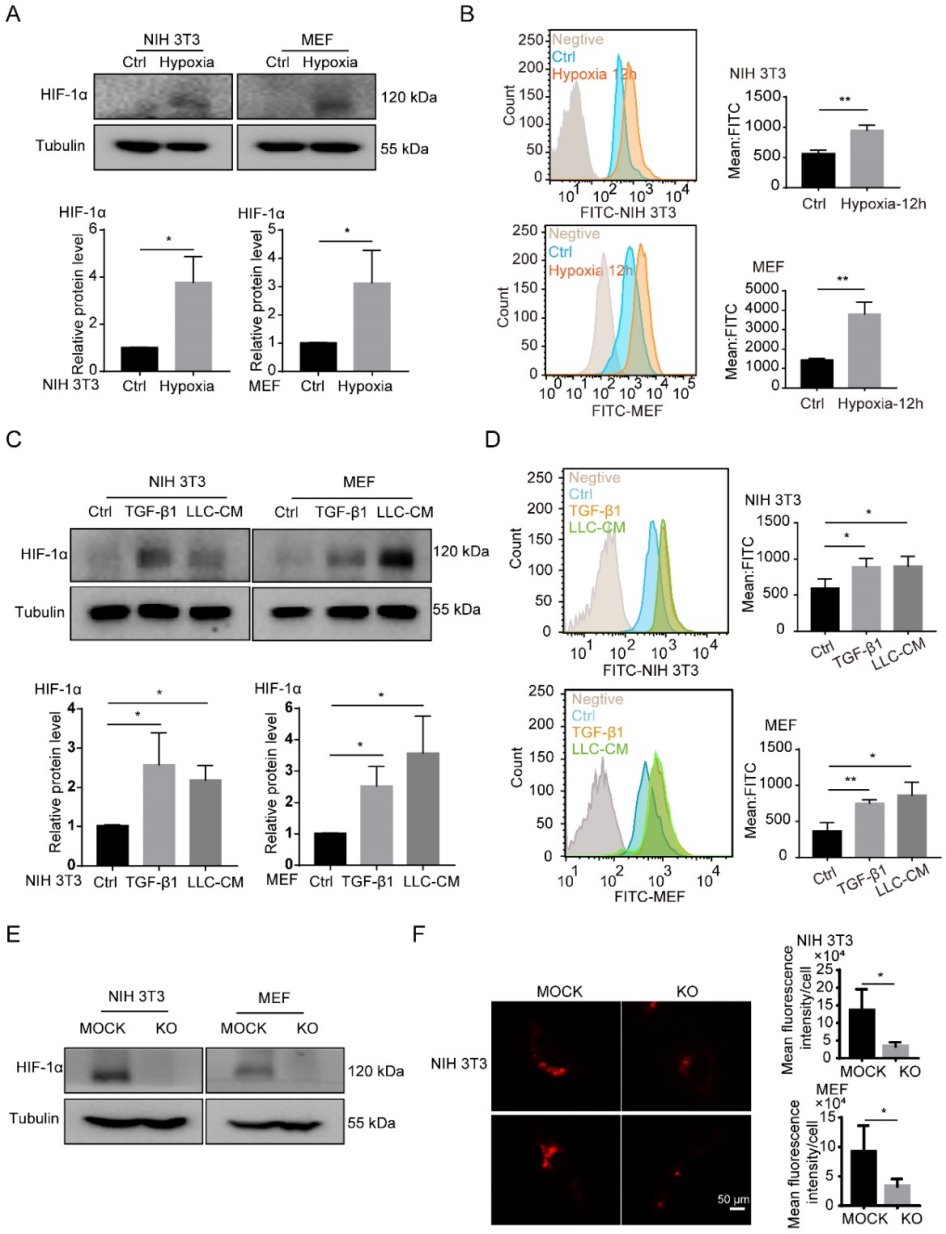
** HIF-1α is necessary for the accumulation of LDs in fibroblasts. (A)** The protein expression of HIF-1α in NIH 3T3 and MEF cells under hypoxia treatment (1% O_2_; N = 3). **(B)** The BODIPY staining of LDs in fibroblasts (NIH 3T3 and MEF cells) which were treated with hypoxia (1% O_2_) for 12h (N = 3). **(C)** The protein expressions of HIF-1α in NIH 3T3 and MEF cells treated with TGF-β1 (2 ng/mL) and LLC conditioned medium (LLC-CM). **(D)** The BODIPY staining of the LDs in the fibroblasts (NIH 3T3 and MEF cells) which were treated with TGF-β1 and LLC-CM (N = 3). **(E)** The protein expressions of HIF-1α were detected in HIF-1α stably knocked out (KO) fibroblasts (NIH 3T3 and MEF cells; N = 3). **(F)** The lipid-tox red staining of the LDs in the HIF-1α MOCK cells (MOCK) and HIF-1α KO cells (KO) (NIH 3T3 and MEF cells; Scale bar: 50 µm; N = 3). Data are shown as the Mean±SD; *p < 0.05, **p < 0.01, ***p < 0.001.

Consistent with the previous data 8, we found that hypoxia, TGF-β1 and tumor CM induced the upregulation of HIF-1α in fibroblasts and the HIF-1α high-expressed fibroblasts was considered as the CAFs (Figures 3A and 3C). Interestingly, a large number of LDs accumulated in activated fibroblasts, which were induced by hypoxia, TGF-β1 and tumor CM (Figures 3B and 3D). Next, to confirm the role of HIF-1α in the accumulation of LDs in fibroblasts, HIF-1α was knocked out (KO) in fibroblasts (NIH 3T3 and MEF cells) (Figures 3E). We observed that HIF-1α deficiency led to an impaired accumulation of LDs in fibroblasts (Figure 3F). These findings suggested that HIF-1α is essential for the accumulation of LDs in fibroblasts.

### HIF-1α promotes the accumulation of LDs in fibroblasts through SCD1

**Figure 4:**
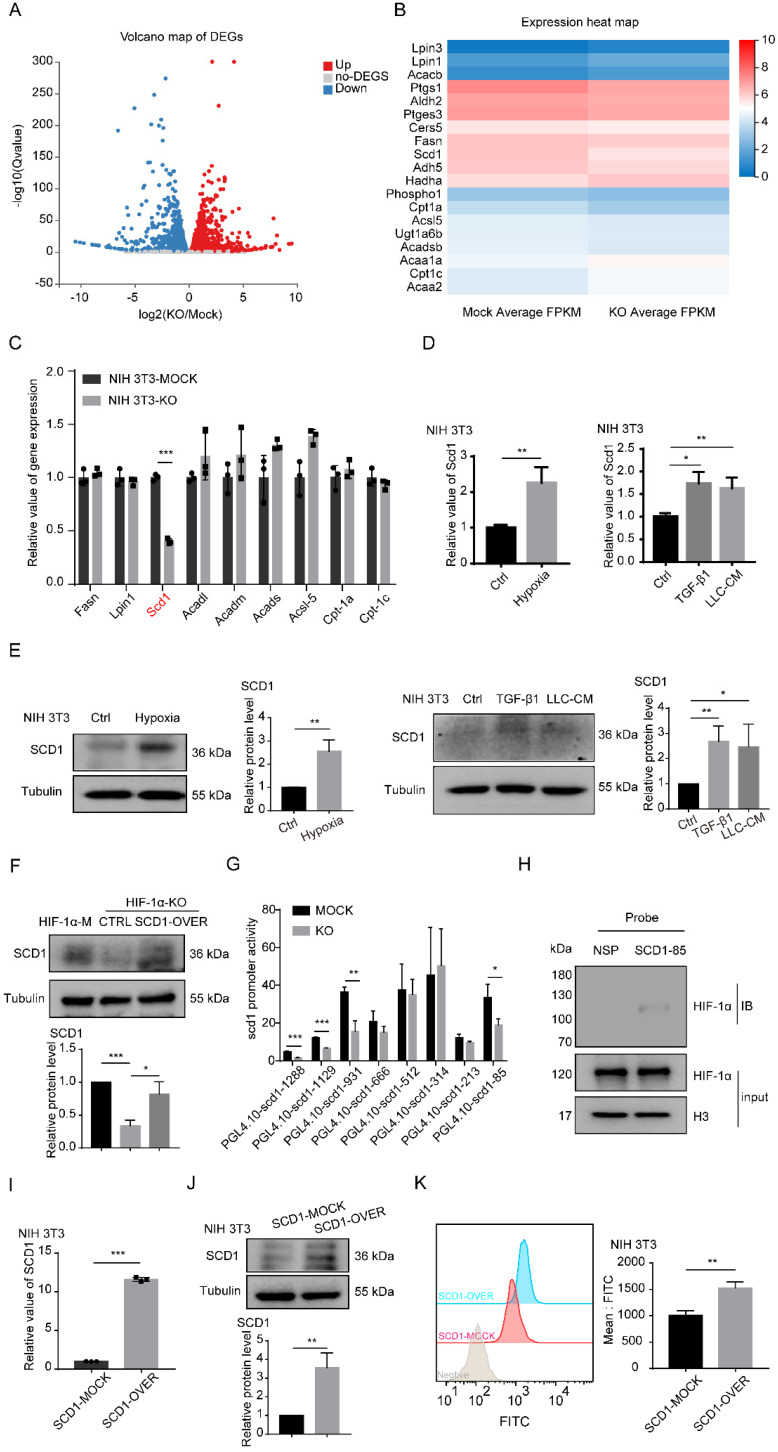
** HIF-1α promotes the accumulation of LDs through SCD1. (A)** The volcano map of gene changes in fibroblasts (NIH 3T3-HIF-1α-MOCK and NIH 3T3-HIF-1α-KO) by RNA-sequencing. **(B and C)** Signature genes involved in lipid metabolism were listed and validated by RT-PCR in fibroblasts (NIH 3T3-HIF-1α-MOCK and NIH 3T3-HIF-1α-KO) (N = 3). **(D and E)** The RNA and protein levels of SCD1 in fibroblasts (NIH 3T3) that were treated with hypoxia (1% O_2_), TGF-β1 and LLC-CM. **(F)** The protein expression of SCD1 in fibroblasts (NIH 3T3-HIF-1α-MOCK and NIH 3T3-HIF-1α-KO cells) transfected with the SCD1-overexpression plasmid. **(G)** The relative promoter activity of mSCD1 in NIH 3T3-MOCK and NIH 3T3-KO cells. **(H)** The expression of bound proteins of HIF-1α in 5'-biotin labeled probes corresponding to the S85 fragment of mSCD1 promoter or a nonspecific probe (NSP) that was incubated with NIH 3T3 cell lysates and streptavidin beads. **(I and J)** The relative expressions of the RNA and protein of SCD1 were determined in the SCD1-MOCK and SCD1-OVER cells (NIH 3T3) (N = 3). **(K)** The BODIPY staining of the LDs in the SCD1-MOCK cells and SCD1-OVER cells (NIH 3T3). Data are shown as the Mean±SD; *p < 0.05, **p < 0.01, ***p < 0.001.

To determine the mechanisms underlying HIF-1α-induced accumulation of LDs in fibroblasts, RNA-sequencing analysis was conducted. The volcano plot showed that 2,978 upregulated genes and 3,389 downregulated genes were differentially detected in HIF-1α-KO fibroblasts (Figure 4A). Most of these genes are related to metabolism, especially involved in lipid synthesis and lipid catabolism (Figures S2A-B and 4B). Then, we performed real-time PCR to validate the characteristic genes. Among these genes, SCD1 was significantly downregulated in NIH 3T3 HIF-1α-KO and MEF HIF-1α-KO cells, while other lipid catabolism genes like Acadm and Cpt-1α were decreased in MEF HIF-1α-KO fibroblasts but not in NIH 3T3 HIF-1α-KO fibroblasts (Figures 4C and S2C). We also observed that the expression of SCD1 was promoted by hypoxia, TGF-β1 and tumor CM (Figures 4D-E, S2D-E). Moreover, decreased SCD1 in HIF-1α KO cells was reversed by the overexpression of SCD1 through vector transfection in fibroblasts (Figures 4F and S2F).

To explore the mechanism of HIF-1α in the expression of SCD1, eight oligonucleotide probes with different lengths in the promoter region of SCD1 were designed (Figure S2G). Those probes were used to investigate the effect of HIF-1α on the SCD1 promoter activity. The luciferase reporter assay showed that KO of HIF-1α significantly decreased the SCD1 promoter activity of NIH 3T3 cells in the promoter region of -1288 ~ +197 bp, -1129 ~ +197 bp, -931 ~ +197 bp and -85 ~ +197 bp (Figure 4G). Next, the activity of the four promoter regions was further examined. In HIF-1α KO fibroblasts, SCD1 promoter activity was obviously inhibited in the promoter region of -1129 ~ +197 bp, -931 ~ +197 bp and -85 ~ +197bp (Figure S2H). As the promoter region of -85 ~ +197 bp was closer to the transcription initiation site, we speculated that HIF-1α might bind to the DNA region. According to the JASPAR database analysis, we predicted that the binding site of the -85 ~ +197 bp region was the most credible promoter binding site and the binding site sequence of SCD1 was 5'-ACGCCT-3'. To ascertain the binding between HIF-1α and the SCD1 promoter, a 5'-biotin-labeled DNA probe for the region of -85 ~ +197 bp of the SCD1 promoter was synthesized, the nuclear protein/DNA complex in NIH3T3 cells was detected using synthesized DNA probe or nonspecific probe (NSP) and their binding was validated by western blot analysis (Figure 4H). These results suggested that HIF-1α can upregulate the expression of SCD1 by regulating the SCD1 promoter activity.

To further investigate the function of SCD1 in fibroblasts, we established stable SCD1-overexpressing cells (NIH 3T3) (Figures 4I and 4J). BODIPY staining showed that more LDs were found in the SCD1-overexpressing cells (Figures 4K and S2K) and the MEF cells overexpressing SCD1 (Figures S2I and S2J). To confirm the relationship between HIF-1α and SCD1 in the accumulation of LDs in fibroblasts, we detected the LDs in fibroblasts with or without SCD1 overexpression under hypoxia and found that SCD1-mock and SCD1-overexpression fibroblasts had increased LDs under hypoxia (Figure S2L). These results indicated that SCD1 is the key downstream gene of HIF-1α that catabolizes the synthesis of LDs in fibroblasts.

### SCD1-overexpressing fibroblasts promote lung cancer growth

**Figure 5:**
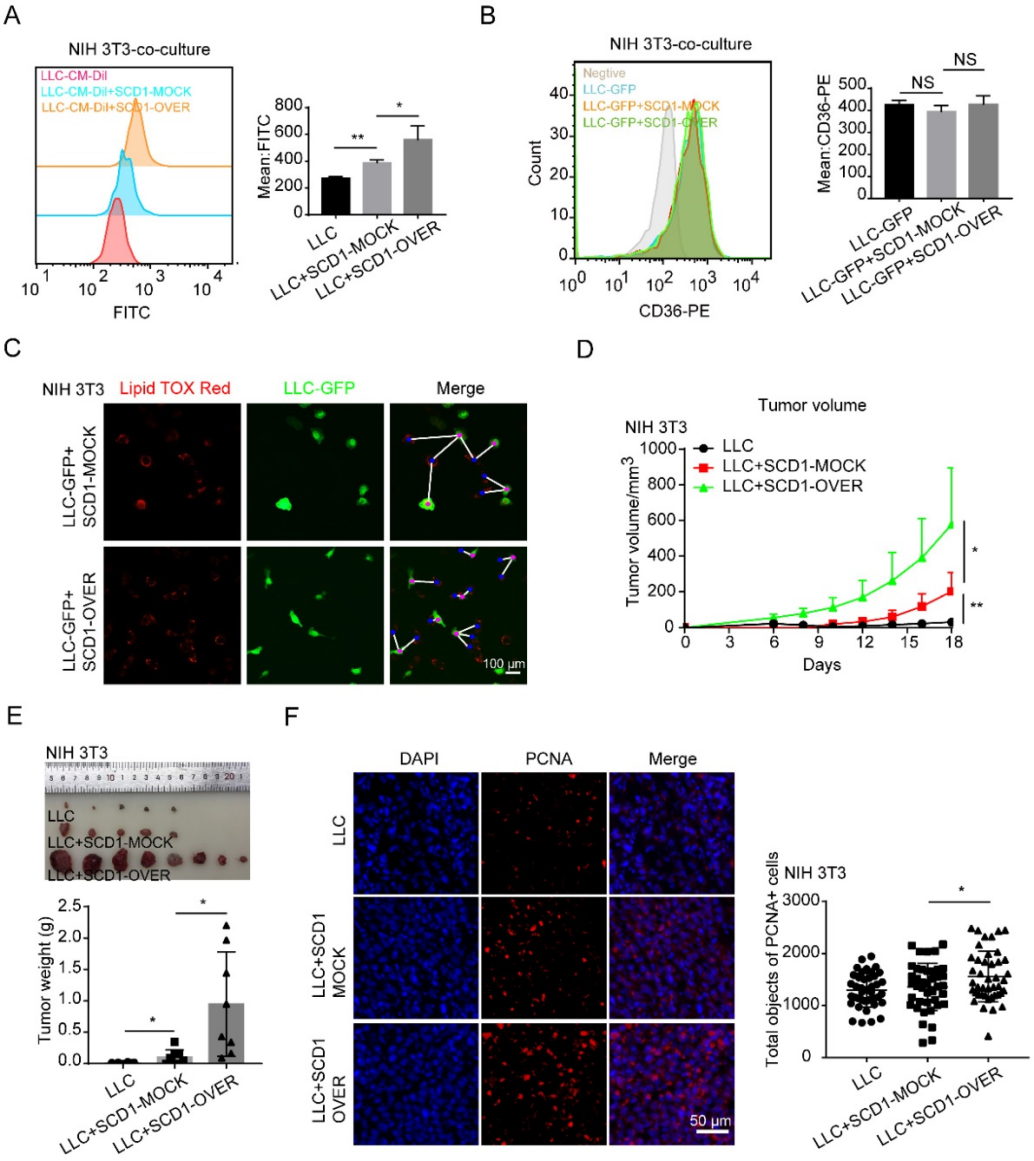
** SCD1-overexpressing fibroblasts promote lung cancer growth. (A and B)** The BODIPY staining and CD36 expression of tumor cells (LLC cells) after co-cultured with or without NIH 3T3-SCD1-MOCK cells and NIH 3T3-SCD1-OVER cells (N = 5). **(C)** The distribution and LD contents of tumor cells (LLC-GFP cells) that were co-cultured with SCD1-MOCK and SCD1-OVER fibroblasts (NIH 3T3) by lipid-tox red staining (Scale bar: 50 µm, N = 3). **(D)** The LLC cells with or without NIH 3T3-SCD1-MOCK cells or NIH 3T3-SCD1-OVER cells were subcutaneously co-injected into the C57 mice (N = 8 mice/group) and the tumor volumes were measured every two days. **(E)** The representative tumor images and tumor weights are presented. **(F)** Representative immunofluorescence images of PCNA (red) in the frozen sections from the tumor tissues (Scale bar: 50 µm). Data are shown as the Mean±SD; NS: *p* ≥ 0.05, *p < 0.05, **p < 0.01.

SCD1, also known as 9-fatty acyl-CoA desaturase, is an endoplasmic reticulum-associated enzyme that catalyzes palmitoyl-CoA and stearoyl-CoA to produce palmitoleic acid (16:1 n-7) and oleic acid (18:1 n-9), which are the main composition of LDs 32. Next, we investigated whether the accumulation of LDs in fibroblasts could affect tumor growth. For this, we established a co-culture system *in vitro*. After co-cultured with tumor cells, fibroblasts overexpressing SCD1 could increase the accumulation of LDs in tumor cells (Figures 5A and S3B). LDs are imported into cells mainly through fatty acid translocase, also known as CD36 33. We found that CD36 expression in tumor cells did not change after they were co-cultured with fibroblasts (Figures 5B and S3D). Interestingly, SCD1-overexpressing fibroblasts localized closer to tumor cells compared with SCD1-mock fibroblasts (Figures 5C and S3C). To further demonstrate whether the increased LDs in SCD1-overexpressing fibroblasts could facilitate lung cancer growth *in vivo*, we established mouse subcutaneous lung cancer models in C57 WT mice. LLC cells were co-injected with or without SCD1-overexpressing fibroblasts subcutaneously into the mice. To eliminate the influence of fibroblasts proliferation on tumor growth, we tested the proliferation of different fibroblasts and found that there was no significant difference between SCD1-mock fibroblasts and SCD1-overexpressing fibroblasts (Figure S3A). However, LLC cells (1×10^5^ cells) were co-injected with fibroblasts and tumor growth was monitored for 20 days, we found that the tumor co-injected with fibroblasts form the subcutaneous graft tumor and tumor co-injected with the SCD1-overexpressing fibroblasts grew faster (Figures 5D and S3E). Additionally, tumor weights increased significantly when SCD1-overexpressing fibroblasts co-injected with LLC cells (Figures 5E and S3F). Furthermore, more PCNA positive cells in the tumor tissues were found when co-injected with SCD1-overexpressing fibroblasts (Figure 5F). These results indicated that the increased accumulation of LDs in fibroblasts promotes lung cancer growth.

### SCD1 blockage inhibits lung cancer progression *in vivo*

**Figure 6:**
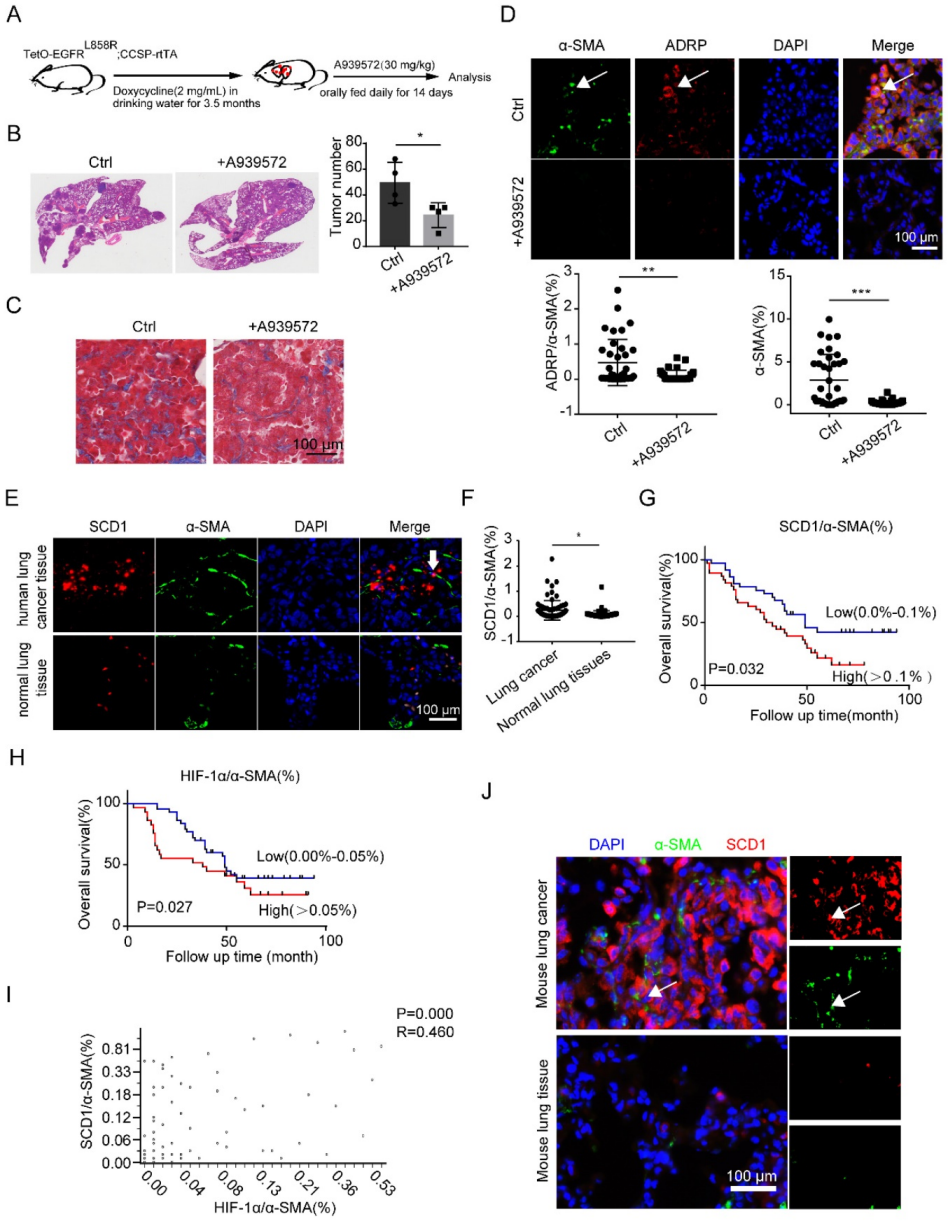
** SCD1 blockage inhibits lung cancer progression *in vivo*. (A)** The procedure of doxycycline-induced mouse lung cancer model (*TetO-EGFR^L858R^; CCSP-rtTA*) treated with A939572 (SCD1 inhibitor: 30 mg/kg) daily for 14 days (N = 4 mice/group). **(B and C)** The H&E and Masson staining of doxycycline-induced mouse lung cancer tissues (*TetO-EGFR^L858R^; CCSP-rtTA*) that were administrated with or without A939572. **(D)** Representative immunofluorescence images of ADRP (red) co-stained with α-SMA (green) in frozen sections of mouse lung cancer tissues (Scale bar: 100 µm). **(E)** Representative immunofluorescence images of SCD1 (red) and α-SMA (green) in the lung adenocarcinoma tissue microarray (Scale bar: 100 µm). **(F)** The statistical analysis of SCD1/α-SMA% expression in lung cancer tissues and normal lung tissues from lung adenocarcinoma tissue microarray data (n = 75). **(G)** Kaplan-Meier overall survival curves for lung adenocarcinoma patients with low (blue line) or high (red line) expression of SCD1 in fibroblasts (co-staining of SCD1 and α-SMA). “Low” indicates that the percentage of co-staining of SCD1 and α-SMA cells is 0.0%-0.1% and “high” indicates the percentage of co-staining of SCD1 and α-SMA cells is > 0.1%. **(H)** Kaplan-Meier overall survival curves for lung adenocarcinoma patients with low (blue line) or high (red line) expression of HIF-1α in fibroblasts (co-staining of HIF-1α and α-SMA). “Low” indicates that the percentage of co-staining of HIF-1α and α-SMA cells is 0.0%-0.05% and “high” indicates that the percentage of co-staining of SCD1 and α-SMA cells is > 0.05%. **(I)** Correlation between co-staining of SCD1 and α-SMA and co-staining of HIF-1α and α-SMA in lung adenocarcinoma paraffin section samples from lung adenocarcinoma tissue microarray (Pearson's correlation test, n = 68, r = 0.460, *p* = 0.000). **(J)** Representative immunofluorescence images of SCD1 (red) co-stained with α-SMA (green) in frozen sections of mouse lung cancer tissue and normal lung tissue (Scale bar: 100 µm). Data are shown as the Mean±SD; *p < 0.05; **p < 0.01; ***p < 0.001.

To monitor the anti-tumor effect of SCD1 inhibitors (iSCD1-A939572) *in vivo*, lung tumor-bearing mice (TetO-EGFR^L858R^; CCSP-rtTA) were treated with vehicle or A939572 starting at 3.5 months after doxycycline was administered (Figure 6A). Remarkably, iSCD1 treatment profoundly impaired doxycycline-induced lung tumor growth, which also contained less collagen in lung cancer tissues (Figures 6B and 6C). Additionally, iSCD1 treatment specifically reduced the accumulation of LDs in CAFs, and α-SMA was also downregulated after the administration of iSCD1 (Figure 6D). These results indicated that the SCD1 might be a novel target involved in the accumulation of LDs in fibroblasts, and targeting SCD1 might be a promising therapeutic strategy to inhibit lung cancer growth.

Finally, we sought to determine whether the observations in mice models could be verified in human lung adenocarcinoma tissues and the matched tumor-adjacent noncancerous lung tissues. We found a higher expression of SCD1 in CAFs than in NFs (Figures 6E and 6F). We further investigated the relationship between the expression level of SCD1 in fibroblasts and the clinical outcomes of lung adenocarcinoma patients using tissue microarrays (n=75). We found that high expression of SCD1 in fibroblasts was significantly associated with short overall survival among lung adenocarcinoma patients (Figure 6G). Consistent with the previous data 8, HIF-1α was highly expressed in lung CAFs (α-SMA%+ cells) (Figures S4A and S4B). The Kaplan-Meier overall survival curves for the expression of HIF-1α/α-SMA% in lung adenocarcinoma patients showed that the expression of HIF-1α in fibroblasts was negatively correlated with the overall survival time (Figure 6H). Interestingly, we found that the expressions of SCD1 and HIF-1α in fibroblasts were positively correlated (Figure 6I). Consistently, SCD1 was also highly expressed in mouse lung CAFs than in NFs (Figure 6J). These results demonstrated that the HIF-1α/SCD1 axis in fibroblasts might affect lung cancer progression, and could be a potential therapeutic target in lung cancer.

## Discussion

It is well-known that overgrowth of tumor cells restricts oxygen diffusion within the tumor, leading to insufficient blood supply to tumor cells and generating a hypoxic microenvironment, which in turn promotes the expression of HIF. HIF-1α has been demonstrated the effectiveness at promoting the formation of tumor blood vessels 34 and tumor metastasis 35. We have previously shown that HIF-1α is essential for the activation of CAFs in lung cancer 8, and we showed here its ability to promote the accumulation of LDs in CAFs. HIF-1α/SCD1 axis contributes to the accumulation of LDs. These results suggested that HIF-1α can be targeted in lung cancer to block the activation of CAFs and provide insights into the mechanism of HIF-1α-induced activation of CAFs.

Although the accumulation of LDs in fibroblasts has been investigated in cancers 36, little is known about the contribution of LDs to the function of fibroblasts in lung cancer. A recent study indicated that unsaturated fatty acids induced the immunosuppressive phenotype of TAMs and promoted tumor growth 19. Kopecka et al. found that the expression and activity of multidrug efflux pumps were facilitated by lipids to modulate the multidrug resistance phenotype in cancer 37. These findings highlighted the complexity of the functions of LDs in different types of cells. In this study, we found that the increased LDs, especially the increased external intake or the synthesis of endogenous fatty acid in fibroblasts could elevate the expression of α-SMA and COL1A2, which indicated the activation of fibroblasts 38, 39. When the formation of LDs was inhibited by the endogenous fatty acid blockers, the activated phenotype of fibroblasts was suppressed. Thus, based on our results, we concluded that the accumulation of LDs induced by the increased synthesis of fatty acids, plays an active role during the phenotypic transformation of fibroblasts. However, considering the heterogeneity of CAFs 2, we cannot yet determine whether there are subgroups of CAFs other than those with the high HIF-1α phenotype that can promote the production of LDs. HIF-1α is involved in the accumulation of LDs through different pathways 21. Our previous work suggested that tumor-promoting fibroblasts could be activated by the upregulation of HIF-1α, which was elevated by hypoxia, TGF-β or tumor CM 6. In this study, we found that the upregulation of HIF-1α promoted the accumulation of LDs in fibroblasts. Consistently, Parathath et al. reported that HIF-1α induced the sterol synthesis and the suppression of cholesterol efflux to increase the sterol content in macrophages of atherosclerotic plaques 35.

As previously mentioned, SCD has a variety of isoforms that play different roles. Human adipose and liver tissues both exhibit significant levels of Scd1 expression. Scd1, Scd2, Scd3, Scd4, Scd5 were primarily expressed in adult mice. SCD5 was also present in chicken and mammals 40. SCD1 is a central lipogenic enzyme that catalyzes the synthesis of monounsaturated fatty acids (MUFA) during lipogenesis in human 41, and it was significantly downregulated after the KO of HIF-1α. SCD1 is known to play an essential role in the progression of cancers 42. Gao et al. found that SCD1 promoted the stemness of ovarian cancer stem cells through the Hippo/YAP pathway 43. Hence, SCD1 is a promising candidate for targeted drug development in STK11/KEAP1 co-mutant lung adenocarcinoma and ameliorated the drug resistance by inducing ferroptosis 44. Wang et al. demonstrated that high expression of SCD1 was associated with the prognosis of gastric cancer patients and might be a therapeutic target in the treatment of gastric cancer 45. SCD1 is also an important biomarker that can improve the survival of lung cancer stem cells, which are stabilized by EGFR to upregulate the synthesis of monounsaturated fatty acids and can promote the growth of lung cancer 46, 47. However, the expression of SCD1 in lung cancer CAFs is still unknown. In the present study, we found that HIF-1α bound to the promoter of SCD1, which in turn promoted the production of lipid droplets in fibroblasts, and these results further established the importance of SCD1 in this process. Because of the differences between the cell lines, besides a reduction in the expression of SCD1, several other lipid catabolism genes like Acadm and Cpt-1α were decreased in the MEF HIF-1α-KO fibroblasts. Therefore, SCD1 is the main downstream gene of HIF-1α that regulates the accumulation of LDs in fibroblasts. We cannot rule out the effects of other lipid metabolism-related genes, and this needs further evidence.

Moreover, we showed that the lung cancer patient survival time was negatively affected by the high expression of SCD1 in fibroblasts. The expression of HIF-1α in lung CAFs was also negatively correlated with the survival time of lung cancer patients, which highlighted that the HIF-1α/SCD1 axis in fibroblasts might be an ideal candidate for predicting the progression of lung cancer. Targeting SCD1 in lung cancer could significantly decrease the LDs in fibroblasts and restrain the formation of collagen in the TME.

Previous studies revealed that CAFs could enhance the production and delivery of ectosomes like lipids to cancer cells compared to that by NFs 48. Moreover, several studies have confirmed the role of aberrant LD formation in promoting tumor cell survival in clear-cell renal cell carcinoma 49, 50 and showed a poor prognosis in high-grade serous carcinoma 51. Our results suggested that the fibroblasts with more LDs were close to the tumor cells in the co-culture system. The number of LDs in tumor cells was elevated after the tumor cells were co-cultured with fibroblasts. It has been reported that the uptake of lipid metabolites secreted from CAFs by colorectal cancer (CRC) cells depends on the expression of CD36 36. However, in this study, the expression of CD36 in tumor cells did not differ between those co-cultured with fibroblasts and those that were not co-cultured with fibroblasts. We speculate that the cell-cell physical contact might be the main reason for the increased lipids in tumor cells that were co-cultured with fibroblasts. However, the detailed mechanism by which the lipid content increases in tumor cells needs further research.

In conclusion, our results established HIF-1α as a master regulator of lipid metabolism in fibroblasts, elevating the abundance of LDs and leading to a tumor-promotion phenotype of lung fibroblasts. This signaling pathway is regulated by the HIF-1α/SCD1 axis. Hence, the HIF-1α/SCD1 axis represents a potential therapeutic target for lung cancer management, along with other components of the lipid metabolism pathway. The findings may have important implications for the therapeutic targeting and development of prognostic markers for lung cancer.

## Supplementary Material

Supplementary figures and tables.

## Abbreviations

CAFs: Cancer associated fibroblasts
LDs: Lipid droplets
TME: Tumor microenvironment
NFs: Normal fibroblasts
iMEFs: Immortalized mouse embryonic fibroblasts
KO: Knock out
α-SMA: α-smooth muscle actin
HIF-1α: Hypoxia inducible factor-1α
FAP: Fibroblast-activating protein
COL1A2: Collagen alpha-2(I) chain
PDPN: Podoplanin
FAS: Fatty acids
TAGs: Triglyceride
ADRP: Adipose differentiation-related protein
SCDs: Stearoyl CoA desaturases
BCAA: branched-chain amino acid
TGF-β1: Transforming growth factor-β1
PCNA: Proliferating cell nuclear antigen
CM: Conditioned medium
LLC: Lewis lung cancer cells
HRP: Horseradish-peroxidase-conjugated
RT-qPCR: Real-time quantitative polymerase chain reaction
DMEM: Dulbecco's modified eagle medium
Cocl2: Cobalt chloride
DGAT: Diglyceride acyltransferase
MAGL: Monoacyglycerol lipase
