# Supplementary material for: Stearoyl-CoA Desaturase-1 dependent lipid droplets accumulation in cancer-associated fibroblasts facilitates the progression of lung cancer
Source: Int J Biol Sci. 2022 Oct 18;18(16):6114–28. doi: 10.7150/ijbs.74924 (PMC9682541; doi:10.7150/ijbs.74924)
Supplement: Supplementary file 1 — Supplementary figures and tables. [file ijbsv18p6114s1.pdf]

Supplementary data

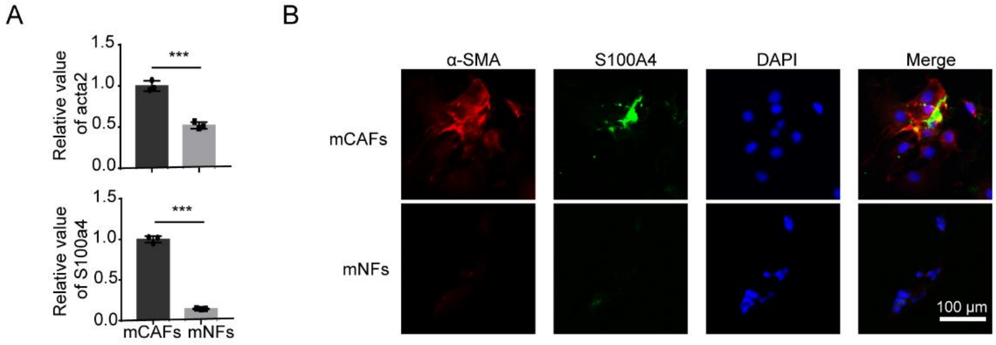

Figure S1

Figure S1. The identification of lung CAFs and NFs

(A) The relative RNA expression of Acta2 and S100a4 in mCAFs and mNFs (N = 3). (B) Representative immunofluorescence images of α-SMA (red) and S100A4 (green) in mCAFs and mNFs (Scale bar: 50 μm). Data are shown as the Mean±SD; ****p* < 0.001.

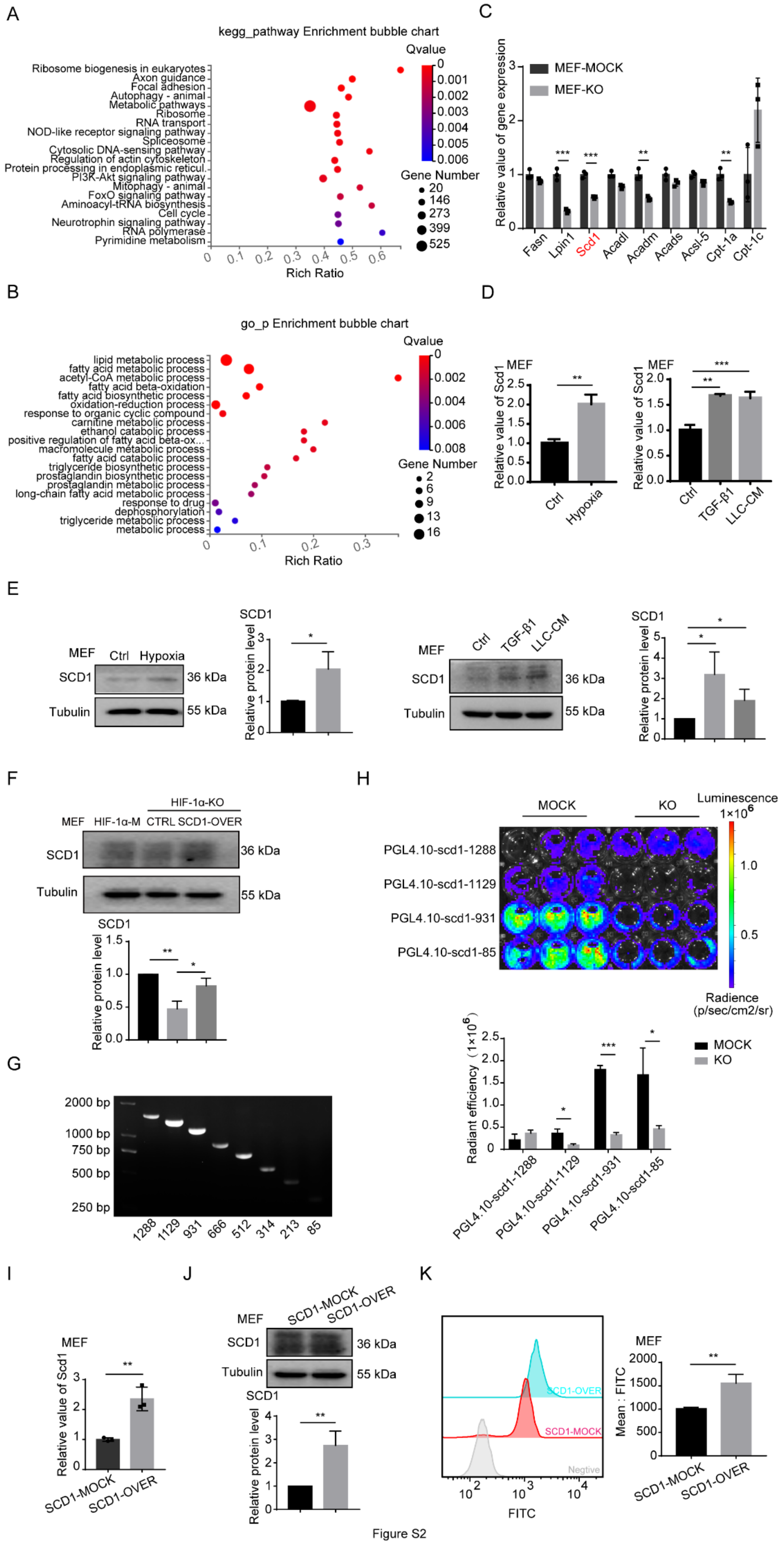

Figure S2

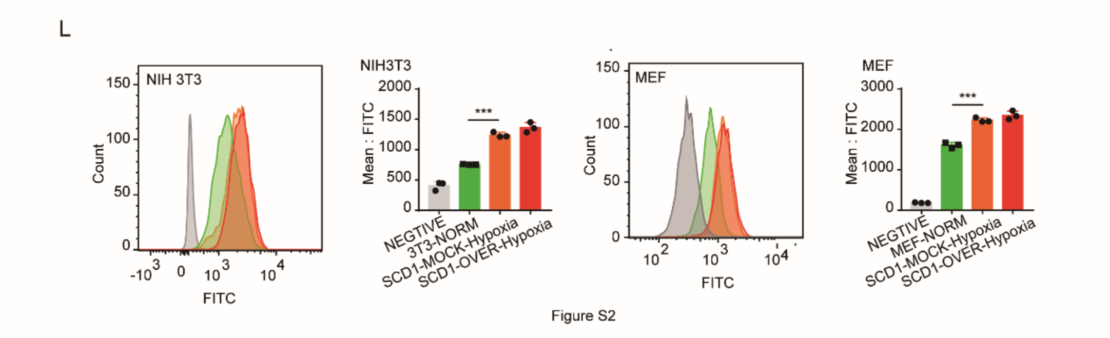

Figure S2. HIF-1α promotes the accumulation of LDs through SCD1

(A) The KEGG pathway analysis for the changes in the genes of fibroblasts (NIH3T3-HIF-1α-MOCK and NIH3T3-HIF-1α-KO). (B) The GO analysis of lipid-associated gene changes in fibroblasts. (C) Signature genes involved in lipid metabolism were validated by RT-PCR in fibroblasts (MEF-HIF-1α-MOCK and MEF-HIF-1α-KO) (N = 3). (D and E) The expression of the gene and protein of SCD1 in fibroblasts (MEF) that were treated with hypoxia, TGF-β1 and LLC-CM. (F) The protein expression of SCD1 in fibroblasts (MEF-HIF-1α-MOCK and MEF-HIF-1α-KO) transfected with the SCD1-overexpression plasmid. (G) The 5'-biotin labeled probes correspond to the different fragments of the mSCD1 promoter (probe 1-8). (H) The luciferase activity of mSCD1 in NIH 3T3-MOCK cells and NIH 3T3-KO cells. (I and J) The relative gene and protein expression of SCD1 that were detected by RT-PCR and western blotting in fibroblasts (MEF-MOCK cells: transfected with SCD1-overexpressed control plasmid; MEF-OVER cells: transfected with SCD1-overexpressed positive plasmid; N = 3). (K) The BODIPY staining of the LDs in MEF-MOCK and MEF-OVER cells. (L) The BODIPY staining of the LDs in SCD1-MOCK and SCD1-OVER fibroblasts under the hypoxia condition for 12h. Data are shown as the Mean±SD; *p < 0.05, **p < 0.01, ***p < 0.001.

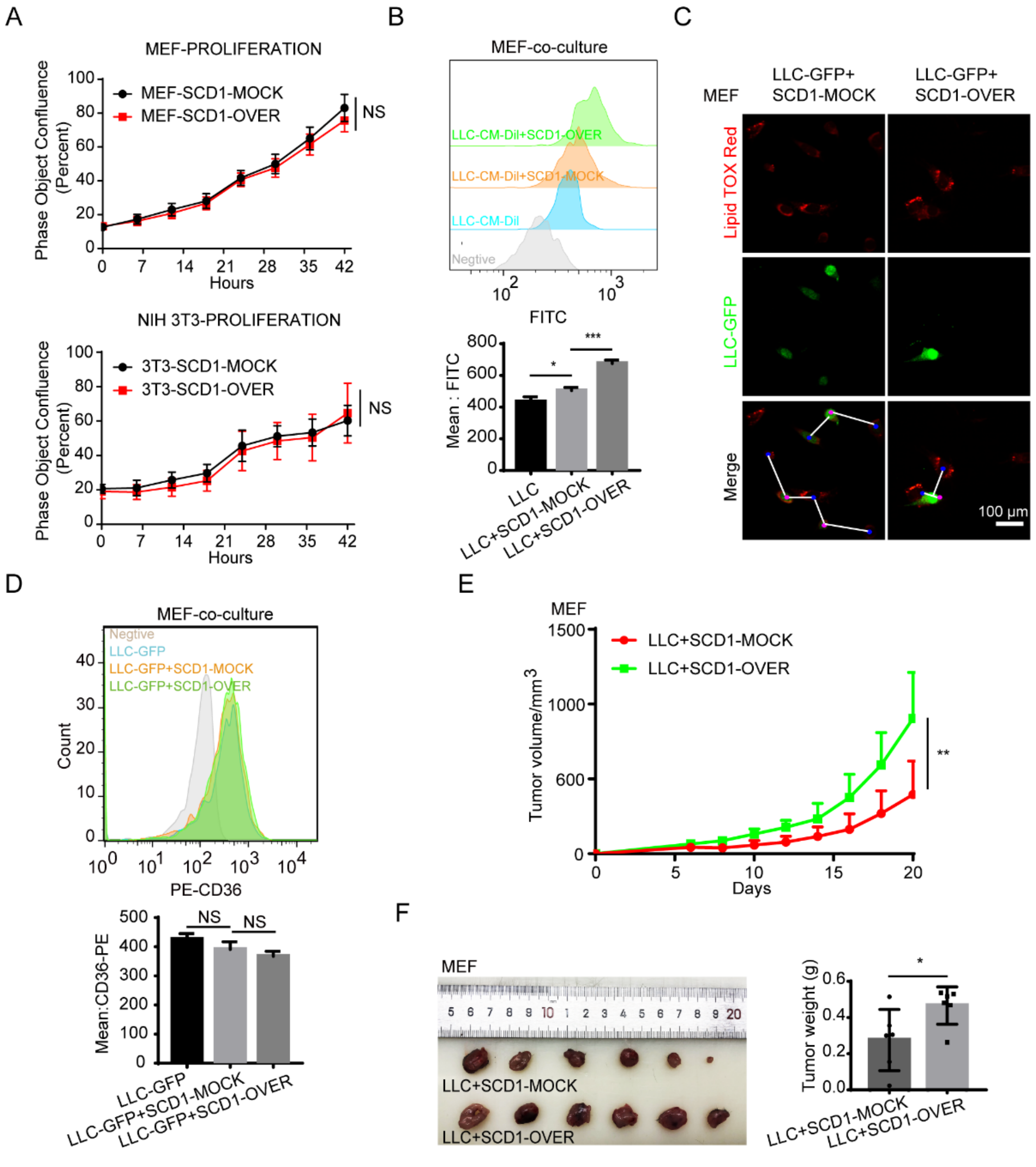

Figure S3

Figure S3. SCD1-overexpressing fibroblasts promote lung cancer growth

(A) The proliferation of fibroblasts (MEF/NIH 3T3-SCD1-MOCK: transfected by viruses with SCD1-overexpressed negative plasmids; MEF/3T3-SCD1-OVER cells: transfected by viruses with SCD1-overexpressed positive plasmids). (B) The BODIPY staining of tumor cells (LLC cells) co-cultured with or without SCD1-MOCK cells and SCD1-OVER cells (MEF cells). (C) The distribution and LD contents of tumor cells (LLC-GFP cells) were detected after the cells were co-cultured with MEF-SCD1-MOCK cells and MEF-SCD1-OVER cells by lipid-tox red staining (Scale bar: 50 μm). (D) Flow cytometry analysis of CD36 expression in tumor cells (LLC-GFP cells) that

were co-cultured with or without MEF-SCD1-MOCK and MEF-SCD1-OVER cells. (E) The different number of LLC cells (1×10^5 cells, 2×10^5 cells and 5×10^5 cells) were subcutaneously co-injected into the C57 mice (n = 3 mice/group), and the tumor volumes were measured every two days. (F) The LLC cells with or without MEF-SCD1-MOCK cells (left) and MEF-SCD1-OVER cells (right) were subcutaneously co-injected into the C57 mice (n = 6 mice/group), and the tumor volumes were measured every two days. (G) The representative tumor images and tumor weights are presented. Data are shown as the Mean±SD; *NS:* $p \geq 0.05$, $^*p < 0.05$, $^{***}p < 0.001$.

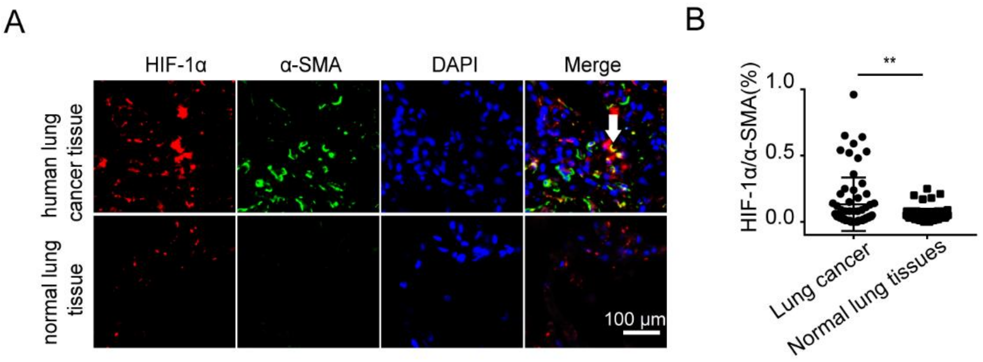

Figure S4

Figure S4. HIF-1α is highly expressed in lung CAFs

(A) Representative immunofluorescence images of HIF-1α (red) and α-SMA (green) in the lung adenocarcinoma tissue microarray (Scale bar: 100 μm). (B) The statistical analysis of HIF-1α/α-SMA% in lung cancer tissues and normal lung tissues from the LC tissue microarray data. Data are shown as the Mean±SD; $^{**}p < 0.01$.

Table S1 The primers in our study are shown as follows

Primer sequence		
S100A4(ms)	Forward(5'-3')	TGAGCAACTTGGACAGCAACA
	Reword(5'-3')	CTTCTTCCGGGGCTCCTTATC
ACTA2(ms)	Forward(5'-3')	CCCAGACATCAGGGAGTAATGG
	Reword(5'-3')	TCTATCGGATACTTCAGCGTCA
18S(ms)	Forward(5'-3')	ACCGCAGCTAGGAATAATGGA
	Reword(5'-3')	CAAATGCTTTCGCTCTGGTC
HIF-1α(ms)	Forward(5'-3')	TCTCGGCGAAGCAAAGAGTC
	Reword(5'-3')	AGCCATCTAGGGCTTTCAGATAA
col1a2(ms)	Forward(5'-3')	TCGTGCCTAGCAACATGCC
	Reword(5'-3')	TTTGTCAGAATACTGAGCAGCAA
scd1(ms)	Forward(5'-3')	TTCTTGCGATACACTCTGGTGC
	Reword(5'-3')	CGGGATTGAATGTTCTTGTCGT
acsl5(ms)	Forward(5'-3')	TCCTGACGTTTGGAACGGC
	Reword(5'-3')	CTCCCTCAATCCCCACAGAC
cpt1a(ms)	Forward(5'-3')	TGGCATCATCACTGGTGTGTT
	Reword(5'-3')	GTCTAGGGTCCGATTGATCTTTG
cpt1c(ms)	Forward(5'-3')	CGGAGACGACGCTTTCGAC
	Reword(5'-3')	CGTAGTTGGAAGTACACCAGGA
acads(ms)	Forward(5'-3')	GACTGGCGACGGTTACACA
	Reword(5'-3')	GGCAAAGTCACGGCATGTC
acadm(ms)	Forward(5'-3')	AACACAACACTCGAAAGCGG
	Reword(5'-3')	TTCTGCTGTTCCGTCAACTCA
acadl(ms)	Forward(5'-3')	TTTCCTCGGAGCATGACATTTT
	Reword(5'-3')	GCCAGCTTTTTCCCAGACCT
lpin1(ms)	Forward(5'-3')	CCTCCGCTCCCGAGAGAAA
	Reword(5'-3')	CGTTGTCTCCCAACTTCATGT
fasn(ms)	Forward(5'-3')	GGAGGTGGTGATAGCCGGTAT
	Reword(5'-3')	TGGGTAATCCATAGAGCCCAG

Table S2 The primers sequence of core promoter region

S-F-1288	CCGCTCGAGGCTGTCCTGGAACTCACCTT
S-F-1129	CCGCTCGAGACACTGGCTAAGCGTGACCA
S-F-931	CCGCTCGAGCCAAGGAATACCTACTGCTCA
S-F-666	CCGCTCGAGGTTGTAACTCAGCGTGTGCT
S-F-512	CCGCTCGAGTGTGAAGTTAGACCGAGTTGTG
S-F-341	CCGCTCGAGCCACAGCAAAGAGGATAAGG
S-F-213	CCGCTCGAGATGACAAGACGGGCTTCAC
S-F-85	CCGCTCGAGTCTTTCCCGTTCTTGGTCC
S-R-197	CGGGGTACCAAACCTGCCCTCCTGACTCT
